# A multidimensional classification of assistive technology for health system integration

**DOI:** 10.3389/fpubh.2026.1875576

**Published:** 2026-07-28

**Authors:** Hitesh K. Sharma, Ruchir Malik, Dhiraj Kumar, Suman Badhal, Ashoo Grover, Rakesh K. Shrivastav, Ravinder Singh

**Affiliations:** 1 Indian Council of Medical Research (ICMR), New Delhi, India; 2 Vardhman Mahavir Medical College (VMMC) and Safdarjung Hospital, New Delhi, India; 3 National Institute of Health and Family Welfare, New Delhi, India

**Keywords:** assistive product, assistive technology, global health, Sustainable Development Goals, Universal Health Coverage

Assistive technology (AT) enables people with functional limitations to maintain or improve daily functioning and participation across the life course, through an ecosystem of devices, products, aids, services and supports (1–4). Yet despite clear relevance to universal health coverage (UHC) and healthy aging, AT remains marginal in many health systems, often managed through welfare or disability schemes rather than as core health technologies (1, 3).

This marginalization is compounded by the way assistive products (APs) are classified and labeled. Across countries, identical or similar products may be treated as “appliances,” “rehabilitation aids,” “consumer goods” or “orthopedic devices,” depending on legacy programmes and professional traditions. Existing international standards such as ISO 9999 provide essential product-level categorization, but they do not systematically relate APs to health-system tiers, financing pathways, user characteristics and equity considerations (5). As a result, policymakers struggle to decide which products to cover, at what level of care, for which users, and with which financing mechanisms (6).

**Figure 1:**
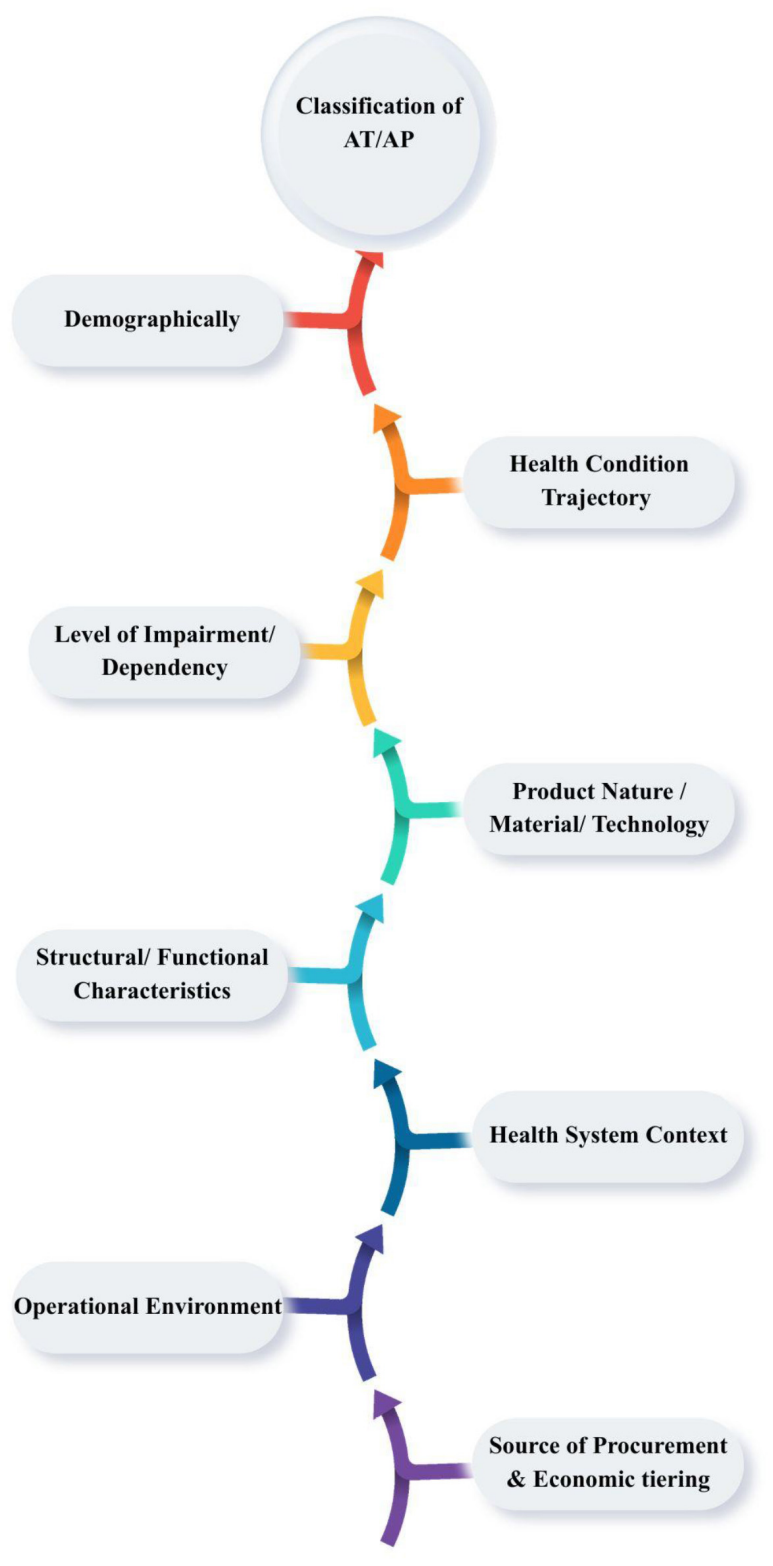
A multidimensional classification framework for assistive technology/assistive products (AT/AP). Due to the complexity and volume of subdimension content, a detailed explanatory classification for each of the seven functional domains is provided in Supplementary Figures S1–S7. Readers are encouraged to refer to the Supplementary material for complete subdimension listings, representative product examples, and domain-specific policy applications.

To address this structural gap, we propose a comprehensive, multidimensional classification framework for AT and APs designed explicitly for health system planning and integration. Rather than a prescriptive taxonomy, it offers an organizing logic that can sit above existing standards and lists, providing a common language for ministries of health, payers, regulators and service provider [Figure 1 and ^**^Figure S1 (See the Supplementary file)].

The dimensions of this framework were derived through a structured process drawing on: (i) a review of existing international and national classification systems (ISO 9999, WHO Priority Assistive Products List, ICF-based frameworks, national essential assistive product lists); (ii) analysis of health system planning frameworks in low-, middle- and high-income countries; and (iii) iterative expert deliberation among the multidisciplinary author team, which includes scientists, rehabilitation professionals and health policy researchers. Although a formal Delphi or systematic review process was beyond the scope of this opinion article, the selection of dimensions reflects convergent evidence from policy mapping, implementation literature and practical health system experience (Table S1).

The framework is anchored in functional domains that are widely recognized in AT practice: mobility, vision, hearing, communication, cognition, self-care and sports and recreations. Within each domain, assistive products are classified to similar depth, reflecting that needs, technologies and service pathways differ substantially across domains and cannot be managed through generic device lists alone. It allows decision-makers to compare, within and across domains, how different products map onto levels of impairment, service platforms and financing options (7).

To our knowledge, no existing framework classifies assistive products simultaneously by functional domain, impairment severity, health-system tier, technology characteristics, user context, and financing and equity dimensions in a way that is directly usable for planning and procurement (Table S1 and Figure S1). We therefore present this as a higher-order structure that can incorporate and connect current standards, rather than replace them.

The framework organizes assistive products (APs) across comprehensive dimensions reflecting real-world use, health system realities and equity. Demographic dimensions include age groups (early childhood 0–5, childhood 6–12, adolescence 13–18, working-age adults 19–59, older adults 60+), gender, regional/cultural contexts (rural, urban, tribal, remote) and socioeconomic access (by HDI category, income level and digital literacy), with additional equity-relevant factors. including disability type, minority or indigenous status, humanitarian settings and long-term maintenance affordability accommodated during national adaptation (Table S2) (8). User and condition-related dimensions capture health condition trajectory (short-term, intermittent, stable long-term, progressive, permanent) and level of impairment/dependency (mild to profound), guiding whether provision should be temporary, recurrent, or lifelong and whether follow-up belongs in primary care or specialist services. Technology and product-related dimensions classify by product nature/technology (mechanical, manual wheeled, powered/electronic, smart/connected, hybrid/advanced) and structural/functional characteristics, distinguishing a walking stick from an AI exoskeleton to anticipate infrastructure, workforce and regulatory needs. Health system and service-related dimensions map APs to primary, secondary, tertiary/specialist services, rehabilitation and community support, plus operational environments (home, education, workplace, public/community spaces, recreation), matching simple products to primary/community settings and complex technologies to tertiary centers. Financing-related dimensions address affordability tiers (no-cost/self-made, low, mid, high, ultra-high-cost) and procurement mechanisms (public provision, social protection, insurance reimbursement, out-of-pocket, subsidies, donations, loan banks), driving equitable planning. Table S2 summarizes all dimensions, sub-categories and their policy applications.

This multidimensional structure moves the field beyond cataloging devices toward understanding systems of care. Because each assistive product can be positioned across the relevant axes, planners can answer practical questions: which products should be available at primary care for people with mild to moderate mobility limitations, in rural areas of low-HDI countries, through public financing; which products belong in secondary-level benefit packages for progressive vision loss; and which high-cost, advanced technologies should be concentrated in tertiary centers with robust maintenance and rehabilitation capacity.

For procurement agencies, the framework helps bundle products not only by type but also by service level, user profile and financing pathway. A country might, for example, prioritize low-cost, mechanically simple mobility and self-care devices for primary-level provision in rural districts, while negotiating pooled purchasing for mid-cost digital hearing aids and communication devices at secondary level and carefully assessing ultra-high-cost exoskeletons or AI-enabled systems through health technology assessment for tertiary centers. For regulators, the classification clarifies where stronger pre-market and post-market surveillance is required, such as for implantable, powered or connected systems, including considerations of data privacy, cybersecurity, interoperability and ethical governance that many low- and middle-income country regulatory systems are not yet equipped to address.

To illustrate operational use, three worked examples span different domains and service levels. A manual wheelchair for a working-age adult with permanent moderate lower-limb impairment in a rural low-HDI setting classifies as: mobility domain → permanent condition → moderate impairment → primary/community level → mechanical technology → low-cost tier → public subsidy financing, signaling decentralized procurement without specialist prescription. A digital hearing aid for an older adult (60+) with progressive mild-to-moderate hearing loss in an urban middle-HDI context maps to: hearing domain → progressive condition → secondary level → powered/electronic technology → mid-cost tier → insurance reimbursement, indicating need for audiological review. An AI-enabled communication device for a child with severe cerebral palsy falls under: communication domain → permanent condition → profound impairment → tertiary/specialist level → smart/connected technology → high-cost tier → health technology assessment required, flagging regulatory review before system-wide adoption. These examples show how the framework generates context-sensitive decision rules for benefit package design, procurement and financing.

The proposed framework is designed to complement, not supplant, existing international and national classification systems and lists. ISO 9999 categories, WHO priority assistive product initiatives, National List of Essential Assistive Products (NLEAP), The Bureau of Indian Standards (BIS), International Classification System of Functioning, Disability and Health (ICF) and various regulatory frameworks can be nested within the multidimensional structure, allowing countries to retain familiar product codes while gaining clarity on where and how these products fit within health-system tiers, financing schemes and equity goals (1, 2, 9). In this sense, the framework provides the “scaffolding” within which diverse AT policies, lists and programmes can be aligned.

By explicitly linking AT to core health-system functions, assessment and prescription, procurement and supply, financing, service delivery and monitoring, the classification reframes AT as an essential health service rather than a discretionary add-on (10). It offers a common reference point for health ministries, insurance agencies, rehabilitation professionals, disabled persons organizations and manufacturers to coordinate around integrated AT provision, enabling practical steps such as mapping existing product lists against framework dimensions, designing essential benefit packages by service level, bundling procurement by user profile and financing tier, and generating equity-sensitive monitoring indicators to track who receives what, where and with what outcome (1, 4).

The path to Universal Health Coverage and the Sustainable Development Goals cannot be completed without assistive technology. Yet across most health systems, AT continues to be treated as a discretionary welfare provision rather than an essential health service, a structural failure with direct consequences for hundreds of millions of people with functional limitations.

The multidimensional framework proposed here is a deliberate response to that failure. By organizing assistive products simultaneously across functional domains, impairment levels, health system tiers, technology types, user demographics, and financing pathways, it offers decision-makers a coherent planning logic that no existing standard currently provides. It does not ask countries to discard what they have built; it gives them a structure within which existing lists, codes and programmes can be aligned and made actionable. Realizing this potential will require addressing implementation barriers including workforce capacity, supply chain development, maintenance systems, financing sustainability, regulatory capacity for advanced technologies, and cross-sectoral coordination between health, social protection and education sectors within which the framework provides an organizing structure, not a ready-made solution.

The test of this framework is not academic. It will be measured by whether ministries of health can use it to design more rational benefit packages, whether procurement agencies can use it to bundle products more equitably, and whether monitoring systems can use it to track who receives what, where, and with what outcome. We acknowledge that this framework has not yet been formally validated, tested in real health systems, or evaluated with persons with disabilities, clinicians, policymakers or procurement stakeholders, limitations that future participatory and implementation research must address. We call on national governments, global health agencies and development partners to adapt and stress-test this framework in diverse country contexts, with meaningful involvement of disabled persons' organizations and end users.
