# Supplementary material for: A multidimensional classification of assistive technology for health system integration
Source: Front Public Health. 2026 Jul 28;14:1875576. doi: 10.3389/fpubh.2026.1875576 (PMC13457714; doi:10.3389/fpubh.2026.1875576)
Supplement: Supplementary file 1 [file Data_Sheet_1.pdf]

A Multidimensional Classification of Assistive Technology for Health System Integration

*Hitesh K Sharma[1†], Ruchir Malik[1], Dhiraj Kumar[1], Suman Badhal[3], Ashoo Grover[1], Rakesh K Shrivastav[2], Ravinder Singh[1]**

[1]*Indian Council of Medical Research (ICMR), Ansari Nagar, New Delhi, India 110029.*

[2]*National Institute of Health and Family Welfare, New Delhi, India 110067.*

[3]*Vardhman Mahavir Medical College (VMMC) and Safdarjung Hospital, New Delhi, India 110029.*

***Corresponding Author:** Dr. Ravinder Singh, Senior Scientist, Indian Council of Medical Research (ICMR), Ansari Nagar, New Delhi - 110029, India

Email: aaressjay@gmail.com

***†Co*-Corresponding Author:** Dr. Hitesh Kumar Sharma, Scientist, Indian Council of Medical Research (ICMR), Ansari Nagar, New Delhi - 110029, India

Email: hiteshs093@gmail.com

Table S1: Comparison of the proposed multidimensional framework with existing assistive technology classification systems across key planning dimensions.

Classification System	Functional Domain	Impairment Level	Health System Tier	Technology Type	User Demographics	Financing/ Equity
ISO 9999	✓	✕	✕	Partial	✕	✕
WHO Priority AP List	Partial	✕	Partial	✕	✕	Partial
ICF-based frameworks	✓	✓	✕	✕	Partial	✕
NLEAP (India)	Partial	✕	Partial	✕	✕	Partial
Proposed Framework	✓	✓	✓	✓	✓	✓

Table S2: Summary of dimensions and sub-dimensions of the proposed multidimensional classification framework for assistive technology, with definitions and policy applications.

Dimension	Sub-categories	Definition	Policy Application
**Functional Domain**	Mobility, Vision, Hearing, Communication, Cognition, Self-care, Sports & Recreation	The primary functional area supported by the assistive product	Guides domain-specific planning and benefit package design
**User Demographics**	Age groups (0–5, 6–12, 13–18, 19–59, 60+); Gender; Setting (rural/urban/remote); HDI category	Characteristics of the intended user population	Ensures equitable coverage across population groups
**Health Condition Trajectory**	Short-term, Intermittent, Stable long-term, Progressive, Permanent	The expected clinical course of the underlying condition	Determines whether provision should be temporary, recurrent or lifelong

**Impairment/Dependency Level**	Mild, Moderate, Severe, Profound	Severity of functional limitation	Links product complexity to appropriate service level
**Technology Type**	Mechanical/manual, Powered/electronic, Smart/connected, Hybrid/advanced	Nature and complexity of the product	Anticipates infrastructure, workforce, safety and regulatory needs
**Health System Tier & Setting**	Primary, Secondary, Tertiary/Specialist, Rehabilitation, Community; Home, Education, Workplace, Public	Level of care and operational environment	Matches products to appropriate service platforms
**Financing & Equity**	Affordability tier (no-cost to ultra-high); Procurement mechanism (public, insurance, OOP, subsidy, loan)	Cost category and funding pathway	Guides reimbursement design, subsidy targeting and procurement strategy

S1(a) Mobility

- **Demographically**
  - **Age**
    - Early Childhood (0-5 Years)
    - Childhood (6-12 Years)
    - Adolescences (13-18 Years)
    - Working-age Adults (19-59 Years)
    - Older Adults (60+ Years)
  - **Gender**
    - Male
    - Female
    - Others
  - **Regional & Cultural Contexts**
    - Rural (Durable wheelchairs, terrain-ready walkers etc.)
    - Urban (Foldable mobility scooters, app-linked tools etc.)
    - Tribal (Traditional posture-supportive walking aids etc.)
    - Remote/Terrain (Lightweight, rugged mobility aids etc.)
    - Cultural Fit (Aids designed for local posture/mobility habits etc.)
  - **Socioeconomic Access by HDI (Human Development Index) Category**
    - Low HDI (Donated crutches, manual wheelchairs etc.)
    - Medium HDI (Community mobility aids, low-cost scooters etc.)
    - High HDI (Powered wheelchairs, GPS-equipped mobility aids etc.)
    - Very High HDI (AI-integrated exoskeletons, smart navigation devices etc.)
- **Health Condition Trajectory**
  - Short-Term Use Products (Post-injury crutches, cast walkers, temporary transfer boards etc.).
  - Intermittent Use Products (Folding walkers or braces for relapsing conditions etc.).
  - Stable Long-Term Use Products (Long-term users of manual wheelchairs or prostheses etc.).
  - Progressive Use Products (Adjustable mobility aids adapting to declining strength or function etc.).
  - Permanent Use Products (Custom power chairs or orthotic-prosthetic systems used lifelong etc.).
- **Level of Impairment/ Dependency**
  - Mild (Independent walking with minimal aids e.g., stick, shoe insert etc.).
  - Moderate (Needs bilateral or intermittent support e.g., crutches, walkers, orthoses etc.).
  - Severe (Cannot ambulate safely without wheeled/powered mobility e.g., manual or powered wheelchair, standing frame etc.).
  - Complete/Profound (Unable to self-mobilise; requires caregiver-assisted or fully supported systems e.g., tilt-in-space chairs, transfer devices etc.).
  - Long-term/Permanent (when applicable) (Ongoing reliance on mobility aids with environmental adaptations etc.).
- **Product Nature / Material/ Technology**
  - Mechanical (Simple non-powered supports such as canes, crutches, walkers or transfer boards).
  - Manual Wheeled (Self or attendant-propelled mobility such as standard or lightweight wheelchairs).
  - Powered/Electronic (Battery-assisted devices including power wheelchairs, scooters or powered walkers).
  - Smart/Connected (Sensor-enabled or AI-assisted systems such as fall-detection aids, navigation supports or adaptive controls).
  - Hybrid/Advanced (Integrated robotic or terrain-adaptive technologies such as exoskeletons or semi-powered gait systems).
- **Structural/ Functional Characteristics**
  - Hardware-Based Systems (Physical assistive devices such as wheelchairs, walkers, standing aids or orthoses).
  - Software-Based Systems (Digital or app-based tools such as navigation, communication or device-control applications.
  - Integrated Systems (Combined hardware and software platforms such as smart or sensor-enabled assistive devices).
  - User-Centred Adaptive Systems (Devices or platforms configurable to individual functional, ergonomic or environmental needs through modular design, personalized fitting or programmable settings).
- **Health System Context**
  - Primary Level (Basic mobility screenings, crutch prescriptions etc.).
  - Secondary Level (Physiotherapist-assisted fitting of walkers or braces etc.).
  - Tertiary / Superspeciality Services (Custom wheelchair seating, gait lab assessments etc.).
  - Rehabilitation & Training (Gait training, mobility skills, device usage education etc.).
  - Community-Based Support (Wheelchair loan banks, NGO-led mobility camps, home adaptation support etc.).
- **Operational Environment**
  - Home (Grab bars, ramps, compact indoor mobility aids).
  - Education (Inclusive school pathways, paediatric seating and classroom mobility supports).
  - Workplace (Accessible workstations, transfer aids, occupational mobility adaptations).
  - Public & Community Spaces (Curb cuts, transport ramps, terrain-adapted mobility devices).
  - Recreation & Leisure (Sports wheelchairs, adaptive cycles, beach or outdoor mobility aids, activity-specific supports).
- **Source of Procurement & Economic tiering**
  - **Procurement & Financing Mechanisms**
    - Indigenous or locally manufactured devices
    - Imported or specialized devices
    - Public provision through health or social protection schemes
    - Insurance or pooled financing reimbursement
    - Out-of-pocket expenditure
    - Subsidies, donations or loan banks
    - Repair, maintenance and lifecycle costs
  - **Cost of the Device/Product/Aid**
    - No-Cost/Self Made (Walking stick, Wooden crutches, PVC-pipe or bamboo walkers etc.).
    - Low-Cost Solutions (Communication cards, printed symbol books, DIY communication boards etc.).
    - Mid-Cost Solutions (Basic speech-generating apps, portable amplifiers etc.).
    - High-Cost Technologies (Eye-tracking AAC systems, dynamic display SGDs etc.).
    - Ultra High-Cost Solutions (BCI-integrated AAC tools, multilingual AI-enhanced predictive devices etc.).

S1(b)Vision

- **Demographically**
  - **Age**
    - Early Childhood (0-5 Years)
    - Childhood (6-12 Years)
    - Adolescences (13-18 Years)
    - Working-age Adults (19-59 Years)
    - Older Adults (60+ Years)
  - **Gender**
    - Male
    - Female
    - Others
  - **Regional & Cultural Contexts**
    - Rural (Durable magnifiers, offline reading tools etc.)
    - Urban (App-integrated vision tools, AI smart glasses etc.)
    - Tribal (Non-textual navigation or symbol-based devices etc.)
    - Remote/Terrain (GPS-integrated canes, solar-powered devices etc.)
    - Cultural (Fit Localized Braille languages, navigation norms etc.)
  - **Socioeconomic Access by HDI (Human Development Index) Category**
    - Low HDI (Basic magnifiers, white canes, tactile markers etc.)
    - Medium HDI (Audio books, affordable screen readers etc.)
    - High HDI (Smart magnifiers, OCR devices, Braille tablets etc.)
    - Very High HDI (AI-based wearables, smart navigation glasses etc.)
- **Health Condition Trajectory**
  - Temporary (Temporary magnifiers, eye shields, basic readers etc.)
  - Episodic (Adjustable lighting tools, adaptive screen filters etc.)
  - Chronic (Standard prescription glasses, static desktop magnifiers etc.)
  - Progressive (Adaptive products; wearables with magnification and text-to-speech; modular assistive systems etc.)
  - Irreversible (Braille devices, screen readers, audio labelling tools, tactile navigation aids etc.)
- **Level of Impairment/ Dependency**
  - Mild Impairment (Reading glasses, font-enhancing or contrast apps etc.)
  - Moderate Impairment (High-powered magnifiers, screen magnifiers, contrast-enhancing devices etc.)
  - Severe Impairment (Audio readers, OCR-based readers, tactile diagrams etc.)
  - Complete/Profound Vision Loss (Braille displays, voice-controlled devices, sensor-based smart canes etc.)
  - Lifelong/Permanent Dependency (Durable Braille tablets, long-term navigation aids, modular AT ecosystems etc.)
- **Product Nature / Material/ Technology**
  - Optical Products/Device/Aid (Hand-held magnifiers, bifocal lenses, telescopic aids etc.)
  - Non-Optical Products/Device/Aid (Contrast mats, bump dots, tactile guides etc.)
  - Electronic/Digital Products/Device/Aid (Video magnifiers, Braille embossers etc.)
  - Wearables (Smart glasses, camera-equipped navigation headgear etc.)
  - Immersive / XR Systems (AR/VR/MR headsets for magnified viewing, spatial audio navigation, orientation training or simulated low-vision rehabilitation)
  - Tactile Tools (Refreshable Braille devices, tactile books and charts etc.)
- **Structural/ Functional Characteristics**
  - Hardware-Based Products (Braille embossers, desktop video magnifiers, portable telescopes etc.)
  - Software-Based Solutions (Audio-reading apps, digital contrast modifiers, AI-assisted navigation or recognition software etc.)
  - Integrated Products (Smartphones or smart wearables loaded with OCR, AI, GPS, screen reading features or AR/VR-based assistive platforms)
  - User-Centred Adaptive Systems (Devices or platforms configurable to individual visual, functional or environmental needs through modular design or personalized settings)
- **Health System Context**
  - Primary care (Vision screening, prescription glasses etc.)
  - Secondary care (Cataract services, provision of low-vision aids etc.)
  - Tertiary / Specialist care (Ophthalmology/optometry services for diagnosis and complex lenses etc.)
  - Specialized Rehabilitation services (Training for low-vision skills, orientation and mobility services, digital/VR-based vision rehabilitation and mobility simulation etc.)
  - Community / Home-based care (Services for the visually impaired, inclusive education programs etc.)
- **Operational Environment**
  - Home Use (Talking kitchen scales, smart lighting systems, tactile appliance overlays etc.)
  - Educational Settings (Portable magnification readers, digital Braille tablets, inclusive testing tools etc.)
  - Workplace Use (Dual-display screen readers, voice-command productivity suites etc.)
  - Public & Community Use (Tactile pavement sensors, audio traffic signals, interactive Braille kiosks, AI- or AR-assisted navigation supports etc.)
  - Recreation & Leisure (Audio-described sports equipment, tactile board games, accessible gaming interfaces, smart navigation or AR/VR-based experiential tools etc.)
- **Source of Procurement & Economic tiering**
  - **Procurement & Financing Mechanisms**
    - Indigenous/locally manufactured/Self made Device/Product/Aid
    - Imported or specialized Device/Product/Aid
    - Public provision through health or social protection schemes
    - Insurance or pooled financing reimbursement
    - Out-of-pocket expenditure
    - Subsidies, donations, loan banks or philanthropic support
    - Repair, maintenance and lifecycle costs
  - **Cost of the Device/Product/Aid**
    - Low-Cost Solutions (Basic crutches, PVC-framed chairs, wooden walkers etc.).
    - Mid-Cost Solutions (Standard manual wheelchairs, basic rollators etc.).
    - High-Cost Solutions (Motorized mobility scooters, tilt/recline power chairs etc.).
    - Ultra High-Cost Solutions (Smart exoskeletons, terrain-tracking powered wheelchairs etc.).

S1(c) Hearing

- **Demographically**
  - **Age**
    - Early Childhood (0-5 Years)
    - Childhood (6-12 Years)
    - Adolescences (13-18 Years)
    - Working-age Adults (19-59 Years)
    - Older Adults (60+ Years)
  - **Gender**
    - Male
    - Female
    - Others
  - **Regional & Cultural Contexts**
    - Rural (Battery-efficient amplifiers, solar-charged aids etc.)
    - Urban (Smart hearing aids, app-controlled tuning etc.)
    - Tribal (Devices compatible with oral/community-specific languages etc.)
    - Remote/Terrain (Durable aids, portable testers etc.)
    - Cultural Fit (Sign language compatibility, stigma-sensitive solutions etc.)
  - **Socioeconomic Access by HDI (Human Development Index) Category**
    - Low HDI (Pocket amplifiers, shared aids, NGO-fitted analog devices etc.)
    - Medium HDI (Basic digital aids, community hearing centers etc.)
    - High HDI (Bluetooth-enabled aids, mobile-linked hearing management etc.)
    - Very High HDI (AI-optimized hearing systems, real-time multilingual captioning etc.)
- **Health Condition Trajectory**
  - Short-Term Use (Temporary amplifiers post-infection or surgery).
  - Intermittent Use (Devices for episodic or fluctuating hearing loss).
  - Stable Long-Term Use (Routine digital hearing aids).
  - Progressive Use (Upgradeable or modular systems for worsening loss).
  - Permanent Use (Implants or lifelong assistive hearing systems).
- **Level of Impairment/ Dependency**
  - Mild (Occasional amplification or sound enhancement for clarity).
  - Moderate (Daily hearing aids for conversational communication).
  - Severe (High-power or directional systems with assistive listening devices).
  - Profound/Complete (Implantable or multimodal auditory-visual supports).
  - Long-term Dependency (Continuous reliance on assistive systems and environmental modifications).
- **Product Nature / Material/ Technology**
  - Mechanical/Passive (Non-powered ear moulds, acoustic horns, sound filters, ear defenders).
  - Body-worn/Basic Amplification (Pocket or wearable analog amplifiers, entry-level aids).
  - Powered/ Digital/Electronic (Digital programmable hearing aids, FM/loop receivers, personal amplifiers).
  - Smart/Connected (Bluetooth/app-controlled, AI-assisted adaptive hearing systems with streaming and noise optimization).
  - Hybrid/Advanced (Implantable or multimodal systems such as cochlear implants, bone-anchored devices or audio-visual-tactile integrated technologies).
- **Structural/ Functional Characteristics**
  - Hardware-Based Systems (Ear-level or tabletop amplification devices).
  - Software-Based Systems (Speech-to-text apps, captioning tools, hearing management software).
  - Integrated Systems (Connected hardware + mobile/cloud platforms).
  - User-Centred Adaptive Systems (Personalized programming, directional focus, noise filtering, remote tuning).
- **Health System Context**
  - Primary Level (Basic screening, OTC/entry-level aids).
  - Secondary Level (Audiometry, programmable hearing aid fitting).
  - Tertiary/Specialist Services (Implant surgery, advanced diagnostics, complex fittings).
  - Rehabilitation & Training (Auditory training, speech therapy, device optimization).
  - Community-Based Support (School programs, hearing camps, peer groups, tele-support).
- **Operational Environment**
  - Home (Amplified phones, TV listeners, vibrating alarms)
  - Education (Classroom FM/loop systems, captioned learning tools)
  - Workplace (Captioned conferencing, assistive microphones, communication aids)
  - Public & Community (Induction loops, visual paging, alerting systems)
  - Recreation & Leisure (Concert filters, adaptive gaming headsets, captioned media tools, sports communication devices)
- **Source of Procurement & Economic tiering**
  - **Procurement & Financing Mechanisms**
    - Indigenous/locally manufactured devices
    - Imported/specialized devices
    - Public provision through health or social schemes
    - Insurance/reimbursement models
    - Out-of-pocket purchase
    - Subsidies/NGOs/loan banks
    - Repair, maintenance and lifecycle costs
  - **Cost of the Device/Product/Aid**
    - Low-Cost (Analog/pocket amplifiers)
    - Mid-Cost (Standard digital hearing aids)
    - High-Cost (Smart/Bluetooth systems)
    - Ultra High-Cost (Implants or AI-enabled advanced systems)

S1(d) Communication

- **Demographically**
  - **Age**
    - Early Childhood (0-5 Years)
    - Childhood (6-12 Years)
    - Adolescences (13-18 Years)
    - Working-age Adults (19-59 Years)
    - Older Adults (60+ Years)
  - **Gender**
    - Male
    - Female
    - Others
  - **Regional & Cultural Contexts**
    - Rural (Durable, low-tech aids, visual cards etc.).
    - Urban (AAC apps, high-tech voice devices etc.).
    - Tribal (Non-written tools using local symbols or gestures etc.).
    - Remote/Terrain (Offline AAC tools, battery-efficient devices etc.).
    - Cultural Fit (Language support, symbol systems reflecting local context etc.).
  - **Socioeconomic Access by HDI (Human Development Index) Category**
    - Low HDI (Manual boards, gesture tools, local AAC aids etc.).
    - Medium HDI (Basic speech apps, school/clinic-provided tools etc.).
    - High HDI (Customized AAC devices, language-specific tablets etc.).
    - Very High HDI (AI voice assistants, dynamic multilingual displays etc.).
- **Health Condition Trajectory**
  - Short-Term Use (Post-surgical or temporary voice/speech supports).
  - Intermittent Use (Devices for episodic speech or language difficulties).
  - Stable Long-Term Use (AAC systems for persistent communication needs).
  - Progressive Use (Modular/upgradeable systems for neurodegenerative conditions).
  - Permanent Use (Lifelong AAC or multimodal communication systems).
- **Level of Impairment/ Dependency**
  - Mild (Occasional supports for clarity, pacing, or articulation).
  - Moderate (Symbol/text-based aids or portable AAC for daily communication).
  - Severe (Dedicated speech-generating or alternative communication devices).
  - Complete/Profound (Non-verbal users requiring fully assisted or multimodal systems).
  - Long-term Dependency (Continuous reliance on AAC with environmental and caregiver support).
- **Product Nature / Material/ Technology**
  - Manual/Low-Tech (Communication boards, alphabet cards, picture books, gesture tools).
  - Device-Based/Basic Electronic/Digital (Portable voice amplifiers, single-message or simple SGD devices).
  - Advanced Electronic/Digital/Software-Based (Tablet or smartphone AAC apps, text-to-speech, predictive keyboards).
  - Smart/Connected (AI-assisted speech generation, cloud-synced vocabulary, context-aware or multilingual systems).
  - Hybrid/Advanced (Eye-tracking, switch-access, gesture-recognition, brain–computer interface or multimodal AAC technologies).
- **Structural/ Functional Characteristics**
  - Hardware-Based Systems (Dedicated SGDs, switches, mounts, adaptive keyboards).
  - Software-Based Systems (AAC apps, predictive language engines, symbol libraries).
  - Integrated Systems (Devices linked with smartphones, IoT or environmental control systems).
  - User-Centred Adaptive Systems (Custom vocabularies, voice selection, personalized layouts and access methods).
- **Health System Context**
  - Primary Level (Basic screening, simple boards or low-tech AAC provision).
  - Secondary Level (Speech-language therapy with device fitting and training).
  - Tertiary/Specialist Services (Advanced AAC evaluation, complex access methods, neuro-rehabilitation).
  - Rehabilitation & Training (User, caregiver and teacher training; AAC literacy development).
  - Community-Based Support (School inclusion programs, peer support groups, home-based services).
- **Operational Environment**
  - Home (Visual schedules, tablet AAC, smart assistants programmed with phrases).
  - Education (Classroom AAC integration, symbol boards, accessible learning tools).
  - Workplace (Text-to-speech software, adaptive keyboards, communication badges).
  - Public & Community (AAC cards, accessible kiosks, service communication supports).
  - Recreation & Leisure (Social communication apps, gaming interfaces, storytelling and creative expression tools).
- **Source of Procurement & Economic tiering**
  - **Procurement & Financing Mechanisms**
    - Indigenous/locally manufactured devices
    - Imported/specialized devices
    - Public provision through health or social schemes
    - Insurance/reimbursement models
    - Out-of-pocket purchase
    - Subsidies/NGOs/loan banks
    - Repair, maintenance and lifecycle costs
  - **Cost of the Device/Product/Aid**
    - No-Cost / Self-made Solutions (Gestures, home signs, handwritten notes, paper alphabet/picture boards, caregiver-mediated interpretation etc.)
    - Low-Cost Solutions (Printed symbol charts, PECS cards, slates/whiteboards, basic voice amplifiers)
    - Mid-Cost Solutions (Tablet AAC apps, text-to-speech software, portable speech devices)
    - High-Cost Solutions (Dedicated SGDs, eye-tracking systems, advanced dynamic display devices)
    - Ultra High-Cost Solutions (BCI-based or AI-integrated communication technologies)

S1(e) Cognition

- **Demographically**
  - **Age**
    - Early Childhood (0-5 Years)
    - Childhood (6-12 Years)
    - Adolescences (13-18 Years)
    - Working-age Adults (19-59 Years)
    - Older Adults (60+ Years)
  - **Gender**
    - Male
    - Female
    - Others
  - **Regional & Cultural Contexts**
    - Rural (Simple visual aids, offline memory tools etc.).
    - Urban (App-based cognitive tools, AI reminders etc.).
    - Tribal (Tools respecting oral traditions, local languages etc.).
    - Remote/Terrain (Low-power, rugged devices etc.).
    - Cultural Fit (Reflect traditional roles, communication styles, beliefs etc.).
  - **Socioeconomic Access by HDI (Human Development Index) Category**
    - Low HDI (Manual boards, gesture tools, local AAC aids etc.).
    - Medium HDI (Basic speech apps, school/clinic-provided tools etc.).
    - High HDI (Customized AAC devices, language-specific tablets etc.).
    - Very High HDI (AI voice assistants, dynamic multilingual displays etc.).
- **Health Condition Trajectory**
  - Short-Term Use Products (Post-injury concussion aids, temporary memory planners, task cue cards).
  - Intermittent Use Products (Exam-time focus aids, episodic seizure-related supports, situational prompts).
  - Stable Long-Term Use Products (Daily planners, reminder watches, structured routine tools).
  - Progressive Use Products (Adaptive supports for dementia, Parkinson's, MS, neurodegenerative disorders).
  - Permanent Use Products (Lifelong supports for intellectual disability, autism, traumatic brain injury).
- **Level of Impairment/ Dependency**
  - Mild (Independent functioning with occasional prompts or checklists)
  - Moderate (Regular reminders or simplified task supports required)
  - Severe (Continuous structured guidance or supervised task completion needed)
  - Complete/Profound (Full caregiver-mediated decision-making and safety support required)
  - Long-term/Permanent Dependency (Ongoing reliance on cognitive aids and environmental adaptations)
- **Product Nature / Material/ Technology**
  - Manual / Low-Tech (Printed schedules, cue cards, calendars, checklists, visual timers, memory notebooks).
  - Basic Electronic (Alarm watches, talking clocks, pill reminders, simple pagers, single-function reminder devices).
  - Digital / Software-Based (Mobile reminder apps, task managers, cognitive training software, tablet or computer-based supports).
  - Smart / Connected (Wearables, GPS trackers, smart-home prompts, cloud-synchronised reminders, context-aware systems).
  - Hybrid / Advanced / AI-Enabled (Integrated smart environments, sensor-based monitoring, adaptive AI cognitive supports, predictive assistance platforms).
- **Structural/ Functional Characteristics**
  - Hardware-Based Systems (Timers, alarms, reminder watches, tracking tags).
  - Software-Based Systems (Apps for memory, task sequencing, digital coaching tools).
  - Integrated Systems (Wearable + smartphone + cloud-based monitoring ecosystems).
  - User-Centred Adaptive Systems (Customizable prompts, simplified interfaces, personalized routines).
- **Health System Context**
  - Primary Level (Screening for cognitive decline, simple planners, caregiver education).
  - Secondary Level (Neuropsychological assessment, structured cognitive aids prescription).
  - Tertiary / Specialist Services (Advanced assistive technologies, dementia support systems, rehabilitation programs).
  - Rehabilitation & Training (Cognitive therapy, executive function training, skills coaching).
  - Community-Based Support (Day-care centers, caregiver support groups, home-based supervision tools).
- **Operational Environment**
  - Home Use (Medication reminders, routine charts, smart kitchen safety prompts).
  - Educational Settings (Visual schedules, task breakdown tools, focus timers).
  - Workplace Use (Digital planners, noise-reduction aids, workflow prompts).
  - Public & Community Use (GPS wayfinding aids, emergency ID systems, orientation supports).
  - Recreation & Leisure (Game-based cognitive trainers, simplified hobby tools, guided activity apps).
- **Source of Procurement & Economic tiering**
  - **Procurement & Financing Mechanisms**
    - Indigenous/locally manufactured devices
    - Imported/specialized devices
    - Public provision through health or social schemes
    - Insurance/reimbursement models
    - Out-of-pocket purchase
    - Subsidies/NGOs/loan banks
    - Repair, maintenance and lifecycle costs
  - **Cost of the Device/Product/Aid**
    - No-Cost / Self-made Solutions (Handwritten schedules, wall charts, labels, color coding, caregiver prompts).
    - Low-Cost Solutions (Printed planners, basic timers, pill organizers, simple alarm devices).
    - Mid-Cost Solutions (Electronic reminder watches, mobile apps, digital planners).
    - High-Cost Technologies (Smart wearables, GPS safety trackers, connected home prompts).
    - Ultra High-Cost Solutions (AI-driven monitoring ecosystems, smart-home automation, research-grade adaptive systems).

S1(f) Self-Care

- **Demographically**
  - **Age**
    - Early Childhood (0-5 Years)
    - Childhood (6-12 Years)
    - Adolescences (13-18 Years)
    - Working-age Adults (19-59 Years)
    - Older Adults (60+ Years)
  - **Gender**
    - Male
    - Female
    - Others
  - **Regional & Cultural Contexts**
    - Rural (Low-tech, durable aids etc.).
    - Urban (Compact, app-based tools etc.).
    - Tribal (Culturally adapted hygiene aids etc.).
    - Remote/Terrain (Lightweight, portable tools etc.).
    - Cultural Fit (Design aligned with local norms and roles etc.).
  - **Socioeconomic Access by HDI (Human Development Index) Category**
    - Low HDI (Manual aids, NGO kits etc.).
    - Medium HDI (Basic electric tools, community training etc.).
    - High HDI (Smart hygiene systems, linked apps etc.).
    - Very High HDI(AI-enabled tools, full digital self-care setups etc.).
- **Health Condition Trajectory**
  - Short-Term Use Products (Post-surgery commode chairs, temporary grab bars, feeding supports).
  - Intermittent Use Products (Fatigue-related or episodic condition supports).
  - Stable Long-Term Use Products (Adaptive utensils, dressing aids, shower chairs).
  - Progressive Use Products (Supports for arthritis, stroke, Parkinson's, dementia).
  - Permanent Use Products (Lifelong self-care assistance for spinal cord injury, cerebral palsy, severe disability).
- **Level of Impairment/ Dependency**
  - Mild (Independent with small adaptations or ergonomic tools).
  - Moderate (Requires assistive aids for selected tasks such as dressing or bathing).
  - Severe (Needs structured devices or partial caregiver assistance for most tasks).
  - Complete/Profound (Fully assisted or caregiver-operated systems required).
  - Long-term/Permanent Dependency (Ongoing reliance on assistive devices and environmental modifications).
- **Product Nature / Material/ Technology**
  - Mechanical/Low-Tech (Long-handled sponges, dressing sticks, adaptive cutlery, raised toilet seats).
  - Manual Support Systems (Shower chairs, commode chairs, transfer benches, grab bars).
  - Powered/Electronic (Electric toothbrushes, automatic feeders, powered grooming devices, adjustable beds).
  - Smart/Connected (Sensor-based fall alerts, smart medication dispensers, digital hygiene reminders, AI-enabled monitoring systems).
  - Hybrid/Advanced (Robotic feeding arms, automated toileting systems, intelligent pressure-relief beds, integrated smart bathrooms).
- **Structural/ Functional Characteristics**
  - Hardware-Based Tools (Bathing seats, rails, utensils, grooming devices)
  - Software-Based Systems (Medication reminder apps, hygiene training tools, caregiver dashboards)
  - Integrated Systems (Smart beds, bathroom safety systems, sensor-based monitoring setups)
  - User-Centred Adaptive Systems (Custom handles, adjustable heights, modular designs tailored to individual ability)
- **Health System Context**
  - Primary Level (Basic ADL screening, low-cost aids, caregiver instruction).
  - Secondary Level (Occupational therapist assessment and fitting of adaptive devices).
  - Tertiary / Specialist Services (Complex positioning systems, pressure care technologies, robotic aids).
  - Rehabilitation & Training (ADL retraining, self-care skill development, home modification guidance).
  - Community-Based Support (Home adaptation programs, NGO hygiene kits, loan banks, caregiver support services).
- **Operational Environment**
  - Home Use (Bathroom adaptations, kitchen aids, dressing supports)
  - Educational Settings (Child-friendly feeding and toileting supports, school hygiene aids)
  - Workplace Use (Accessible washrooms, ergonomic self-care adaptations)
  - Public & Community Use (Accessible toilets, hygiene stations, transport-friendly aids)
  - Recreation & Leisure (Portable hygiene kits, travel commodes, camping/bathing adaptations for outdoor activities)
- **Source of Procurement & Economic tiering**
  - **Procurement & Financing Mechanisms**
    - Indigenous/locally manufactured devices
    - Imported/specialized devices
    - Public provision through health or social schemes
    - Insurance/reimbursement models
    - Out-of-pocket purchase
    - Subsidies/NGOs/loan banks
    - Repair, maintenance and lifecycle costs
  - **Cost of the Device/Product/Aid**
    - No-Cost / Self-made Solutions (Improvised grab rails, modified spoons, homemade bathing stools, cloth straps).
    - Low-Cost Solutions (Basic adaptive utensils, plastic commodes, dressing aids).
    - Mid-Cost Solutions (Standard shower chairs, transfer benches, adjustable supports).
    - High-Cost Technologies (Powered beds, electric feeders, advanced grooming devices).
    - Ultra High-Cost Solutions (Robotic feeding systems, automated toileting systems, smart integrated bathrooms).

S1(g) Sports & Recreation

- **Demographically**
  - **Age**
    - Early Childhood (0-5 Years)
    - Childhood (6-12 Years)
    - Adolescences (13-18 Years)
    - Working-age Adults (19-59 Years)
    - Older Adults (60+ Years)
  - **Gender**
    - Male
    - Female
    - Others
  - **Activity / Sport Contexts**
    - Recreational & Leisure Play (Home games, hobby activities, light fitness tools etc.)
    - School & Community Sports (Inclusive playgrounds, adapted PE and community sports equipment etc.)
    - Competitive / Para-Sports (Sports wheelchairs, racing prostheses, handcycles, performance gear etc.)
    - Fitness & Exercise (Adaptive gym machines, resistance trainers, balance and strengthening devices etc.)
    - Outdoor & Adventure (All-terrain recreation chairs, trekking aids, beach or trail mobility devices etc.)
    - Aquatic & Water Sports (Swim supports, flotation aids, pool access and transfer devices etc.)
    - Virtual / Digital Recreation (Exergaming systems, VR/AR-based training or therapeutic gaming platforms etc.)
  - **Socioeconomic Access by HDI (Human Development Index) Category**
    - Low HDI (Community-made play aids, low-cost adaptive equipment etc.)
    - Medium HDI (Standard adaptive sports kits, shared recreational devices etc.)
    - High HDI (Specialized adaptive sports gear, performance equipment etc.)
    - Very High HDI (Smart wearables, AI-assisted coaching tools, robotic/powered technologies etc.)
- **Health Condition Trajectory**
  - Short-Term Use Products (Post-injury supports, temporary braces, activity recovery aids etc.)
  - Intermittent Use Products (Devices for episodic fatigue, pain or functional limitation etc.)
  - Stable Long-Term Use Products (Daily-use adaptive recreation equipment etc.)
  - Progressive Use Products (Adjustable/adaptive devices for declining strength or endurance etc.)
  - Permanent Use Products (Customized lifelong sports or leisure assistive systems etc.)
- **Level of Impairment**
  - Mild Impairment (Jar openers, nonslip mats, electric toothbrushes etc.).
  - Moderate Impairment (Sock aids, long-handled sponges, adapted cutlery etc.).
  - Severe Impairment (Powered grooming tools, voice-activated self-care devices etc.).
  - Total Assistance Required (Devices integrated with caregiving, e.g., sensor-equipped transfer slings, automated wash toilets etc.).
  - Permanent Dependency (Consistent use of full assistive setups, e.g., self-care systems for high-level spinal cord injury) etc.).
- **Product Nature / Material/ Technology**
  - Mechanical (Modified bats/rackets, grip aids, balance supports, adapted cycles etc.)
  - Manual Adaptive Equipment (Sports wheelchairs, handcycles, sledges, adaptive trikes etc.)
  - Powered/Electronic (Power-assist cycles, motorized recreation chairs, electronic training devices etc.)
  - Smart/Connected (Sensor-enabled wearables, performance trackers, app-linked adaptive equipment etc.)
  - Hybrid/Advanced (Robotic trainers, exoskeleton-assisted sports systems, AR/VR-based interactive platforms etc.)
- **Structural/ Functional Characteristics**
  - Hardware-Based Tools (Physical adaptive sports gear, protective supports, transfer aids etc.)
  - Software-Based Systems (Fitness apps, virtual coaching, accessible gaming interfaces etc.)
  - Integrated Systems (Smart equipment combining sensors, software and feedback mechanisms etc.)
  - User-Centred Adaptive Systems (Adjustable, modular or customizable devices tailored to individual ability and sport type)
- **Health System Context**
  - Primary Level (Basic activity screening, provision of simple recreational aids).
  - Secondary Level (Physiotherapy or occupational therapy–guided adaptive sports prescription).
  - Tertiary / Specialist Services (Sports rehabilitation centres, prosthetic/orthotic customization, performance training clinics).
  - Rehabilitation & Training (Skill-building, conditioning, adaptive sports training programs).
  - Community-Based Support (Inclusive sports clubs, NGO camps, recreational participation programs).
- **Operational Environment**
  - Home Use (Indoor exercise tools, hobby adaptations, light recreational devices)
  - Education Settings (Inclusive playgrounds, adaptive PE kits, school sports modifications)
  - Workplace & Wellness Spaces (Accessible gyms, employee fitness adaptations, recreational clubs)
  - Public & Community Spaces (Parks, stadiums, trails, beaches with adaptive access equipment)
- **Source of Procurement & Economic tiering**
  - **Procurement & Financing Mechanisms**
    - Indigenous/locally manufactured devices
    - Imported/specialized devices
    - Public provision through health or social schemes
    - Insurance/reimbursement models
    - Out-of-pocket purchase
    - Subsidies/NGOs/loan banks
    - Repair, maintenance and lifecycle costs
  - **Cost of the Device/Product/Aid**
    - No-Cost / Self-made Solutions (Improvised grab rails, modified spoons, homemade bathing stools, cloth straps).
    - Low-Cost Solutions (Basic adaptive utensils, plastic commodes, dressing aids).
    - Mid-Cost Solutions (Standard shower chairs, transfer benches, adjustable supports).
    - High-Cost Technologies (Powered beds, electric feeders, advanced grooming devices).
    - Ultra High-Cost Solutions (Robotic feeding systems, automated toileting systems, smart integrated bathrooms).

Figure S1. Multi-dimensional classification framework for assistive technology needs across seven functional domains. Each panel presents a structured taxonomy of assistive products organised across common classification dimensions, including health condition trajectory, demographic factors (age, gender), regional and cultural contexts, socioeconomic access by Human Development Index (HDI) category, operational environment, product nature and technology type, structural and functional characteristics, health system level of care, level of impairment and dependency, and procurement and financing mechanisms (cost tiering and source of supply) applied respectively to: **(a) Mobility**, encompassing aids from basic canes to AI-integrated exoskeletons; **(b) Vision**, spanning optical magnifiers to immersive extended reality (XR) navigation systems; **(c) Hearing**, ranging from passive ear moulds to cochlear implants and AI-optimised hearing systems; **(d) Communication**, covering low-tech symbol boards to brain–computer interface augmentative and alternative communication (AAC) devices; **(e) Cognition**, including printed schedules and pill reminders through to AI-driven smart-home monitoring ecosystems; **(f) Self-Care**, addressing adapted utensils and grab bars to robotic feeding and automated toileting systems; and **(g) Sports & Recreation**, encompassing modified grip aids to exoskeleton-assisted and AR/VR-based adaptive sports platforms. Across all domains, products are further stratified by cost tier (no-cost/self-made to ultra-high-cost solutions) and financing pathway (public provision, insurance reimbursement, out-of-pocket expenditure, subsidies, and lifecycle maintenance costs).
